# Finite element study of the biomechanical effects on the rotator cuff under load

**DOI:** 10.3389/fbioe.2023.1193376

**Published:** 2023-06-27

**Authors:** Zhengzhong Yang, Guangming Xu, Jiyong Yang, Xiaosheng Lin

**Affiliations:** ^1^ Department of Orthopaedics, Shenzhen Pingle Orthopedic Hospital and Shenzhen Pingshan Traditional Chinese Medicine Hospital, Affiliate Guangzhou University of Chinese Medicine, Shenzhen, Guangdong, China; ^2^ Department of Orthopaedics, Shenzhen Hospital of Integrated Chinese and Western Medicine, Guangzhou University of Chinese Medicine, Shenzhen, Guangdong, China; ^3^ Department of Orthopedics, The Fifth Clinical College of Guangzhou University of Chinese Medicine, Guangzhou, Guangdong, China

**Keywords:** supraspinatus, infraspinatus, abduction, adduction, stress

## Abstract

Rotator cuff injuries account for 50% of shoulder disorders that can cause shoulder pain and reduced mobility. The occurrence of rotator cuff injury is related to the variation in shoulder load, but the mechanical changes in the rotator cuff caused by load remain unclear. Therefore, the mechanical results of the rotator cuff tissue during glenohumeral abduction and adduction were analyzed based on a finite element shoulder model under non-load (0 kg) and load (7.5 kg) conditions. The results showed that the maximum von Mises stress on the supraspinatus muscle was larger than that on the subscapularis, infraspinatus, and teres minor muscles during glenohumeral abduction. Compared with the non-load condition, the maximum von Mises stress on the supraspinatus muscle increased by 75% under the load condition at 30° abduction. Under the load condition, the supraspinatus joint side exhibited an average stress that was 32% greater than that observed on the bursal side. The von Mises stress on the infraspinatus muscle was higher than that in other rotator cuff tissues during adduction. The stress on the infraspinatus muscle increased by 36% in the load condition compared to the non-load condition at 30° adduction. In summary, the increased load changed the mechanical distribution of rotator cuff tissue and increased the stress differential between the joint aspect and the bursal aspect of the supraspinatus tendon.

## 1 Introduction

Chronic shoulder pain ranks the third most prevalent musculoskeletal pain, following chronic headache and chronic low back pain (Noten et al., 2017). Rotator cuff tears account for 50% of shoulder injuries (Gigliotti et al., 2017), which can cause shoulder pain and limited mobility. Epidemiological studies showed that rotator cuff injury had a prevalence of 31% in people aged 60–69 years and an estimated 65% in people aged over 80 years (Doiron-Cadrin et al., 2020). The use of load-bearing exercises is often recommended for patients with rotator cuff injury to improve shoulder muscle mass and reduce pain (Kjær et al., 2018; Macías-Hernández et al., 2021). However, continuous and repetitive stress leads to muscular imbalance and degeneration of the rotator cuff tissue (Rodriguez Diez-Caballero et al., 2020; Coulet et al., 2022). The research (Rodriguez Diez-Caballero et al., 2020) found that repetitive load-bearing between 3 and 15 kg was one of the occupational risk factors for chronic injury to the shoulder tissue. Cheema et al. (2007) reported an elderly patient suffering a rotator cuff tear during progressive resistance training. Therefore, it is necessary to study the internal mechanical changes in the rotator cuff tissue under shoulder load conditions. Calver et al. (2022) examined supraspinatus and infraspinatus muscle activation patterns in different postures by electromyography. Hecker et al. (2021) analyzed the overall muscle strength of the shoulder during upper extremity activity by electronic isometric force dynamometry. However, electromyography does not reflect specific muscle forces, and it is difficult to analyze local muscle forces using a dynamometer. At present, the stress distribution in the rotator cuff tissue during shoulder activity remains unclear.

Shoulder studies have widely used finite element analysis to analyze shoulder movement, fractures, and surgical implantation (Islán Marcos et al., 2019; Chen et al., 2020). Previous finite element analyses of rotator cuff injuries mainly focused on the mechanical changes in the supraspinatus muscle (Quental et al., 2016; Quental et al., 2020). Inoue et al. (2013) analyzed the effect of abduction of the glenohumeral joint on the supraspinatus muscle under non-load conditions. Islán Marcos et al. (2019) analyzed the stress on shoulder bone and cartilage during glenohumeral motion but did not explore the stress distribution of the rotator cuff tissue in detail. Recently, Guo et al. (2022) constructed a finite element shoulder model containing rotator cuff tissue and analyzed the changes in rotator cuff muscle stress distribution induced by different recurve bow movements. However, the biomechanical effects of load changes on the rotator cuff tissue have not been investigated clearly.

Therefore, this study analyzed the mechanical effects on rotator cuff tissue under non-load and load conditions based on a finite element shoulder model and discussed the mechanical distribution and stress concentration sites of the rotator cuff tissues. First, the loading conditions under non-load and load conditions were simulated on the shoulder finite element model. Second, mechanical loads were applied to simulate the abduction and adduction movements of the glenohumeral joint. We then analyzed the effects of mechanical changes under different loads on the rotator cuff tissue during abduction and adduction.

## 2 Materials and methods

### 2.1 Geometry reconstruction

**FIGURE 1:**
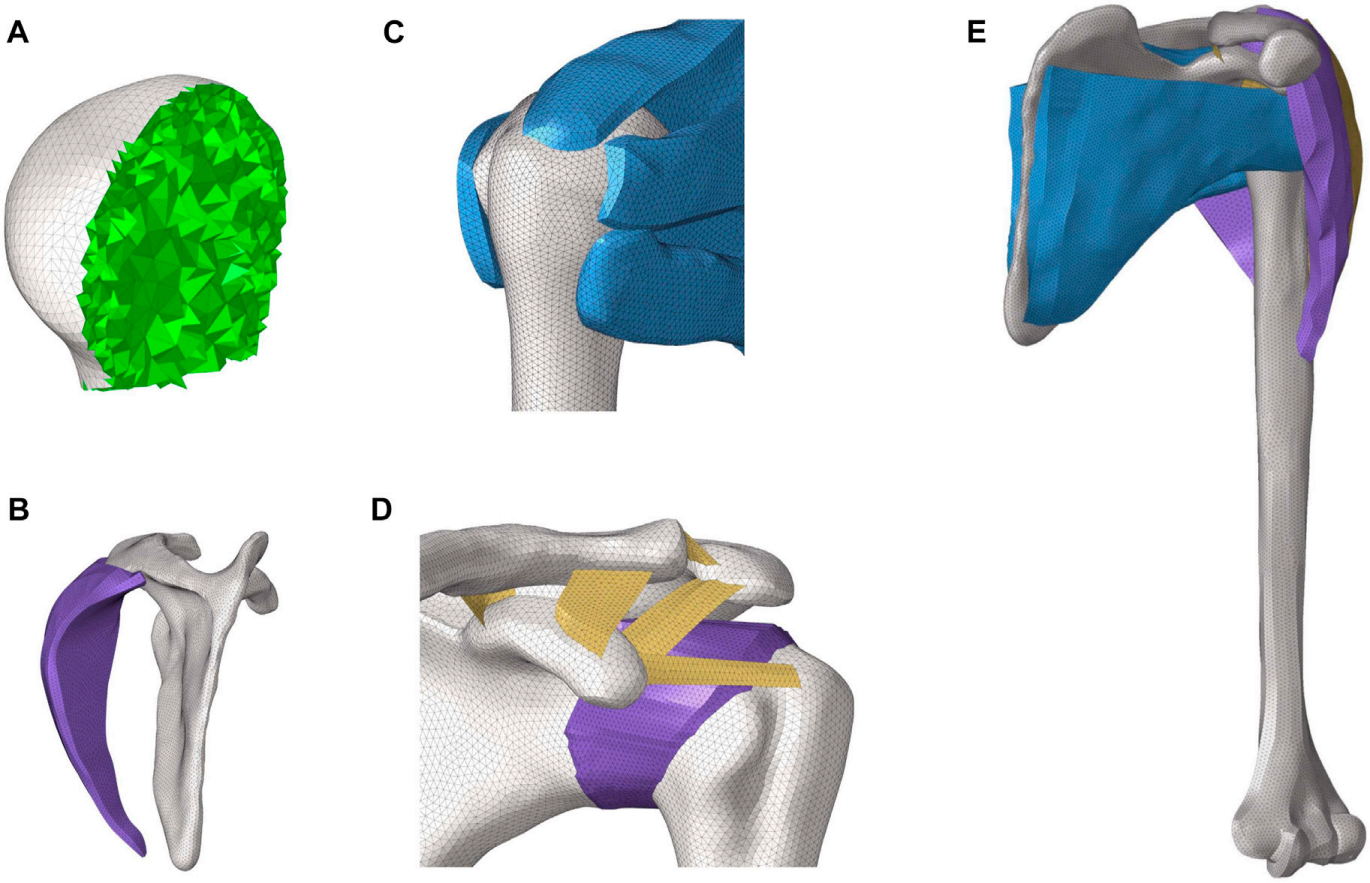
3D finite element model of the shoulder joint. **(A)** Internal structure of the humeral head. **(B)** Lateral view of the posterior deltoid and scapula. **(C)** Construction of rotator cuff tissue. **(D)** Composition and distribution of ligaments. **(E)** Whole model of the shoulder joint.

The image data from a healthy male volunteer (age, 30) were used to construct a finite element shoulder model. The model was constructed as described previously (Yang et al., 2023). The finite element shoulder model consisted of rotator cuff tissue, joint capsule, cortical bone, cancellous bone, joint capsule, deltoid muscle, ligaments, and articular cartilage in Figure 1. The mesh size of the cortical bone, rotator cuff, and deltoid muscle was set at 1 mm with first-order triangular elements. The cancellous bone was a solid structure formed by filling the cortical bone. First-order tetrahedral elements were applied to simulate the cancellous bones. The cortical bone and cancellous bone were made of isotropic elastic material. The joint capsule formed a dense annular package with the humeral head and glenoid. Isotropic hyperelastic material was used for the joint capsule. The ligaments included the glenohumeral ligament, acromioclavicular ligament, and coracoclavicular ligament. Ligaments and joint capsule were modeled with first-order triangular elements. The rotator cuff tissue was divided into supraspinatus, subscapularis, teres minor, and infraspinatus tissues, while the deltoid muscle was divided into anterior, middle, and posterior deltoid muscles. The aforementioned muscles were simulated as non-linear hyperelastic and incompressible (neo-Hookean) (Dao et al., 2014). The material properties of each component are summarized in Table 1 (Koh et al., 2004; Duprey et al., 2007).

**TABLE 1:** Material properties of the components.

Component	Material type	*p* (kg/L)	Material parameter
Cortical bone			
Humerus	Isotropic elastic	2	E = 15,000 MPa; *ν* = 0.3
Scapula	Isotropic elastic	2	E = 15,000 MPa; *ν* = 0.3
Clavicle	Isotropic elastic	1.8	E = 17,000 MPa; *ν* = 0.3
Cancellous bone	Isotropic elastic	1.5	E = 1,000 MPa; *ν* = 0.3
Capsule	Isotropic hyperelastic	1	C1 = 0.27; C2 = 4.4
Muscle			
Supraspinatus	Non-linear hyperelastic and incompressible (neo-Hookean)	1	
Infraspinatus			
Teres minor			
Subscapularis			
Deltoid muscle			
Ligament			
Acromioclavicular ligament	Non-linear elastic	1	E = 10.4 MPa; *ν* = 0.3
Coracoclavicular ligament		1	E = 9.6 MPa; *ν* = 0.3
Glenohumeral ligament		1	E = 150 MPa; *ν* = 0.3

E, Young’s modulus; *ν*, Poisson’s coefficient; *p*, material density.

### 2.2 Mesh convergence test

**FIGURE 2:**
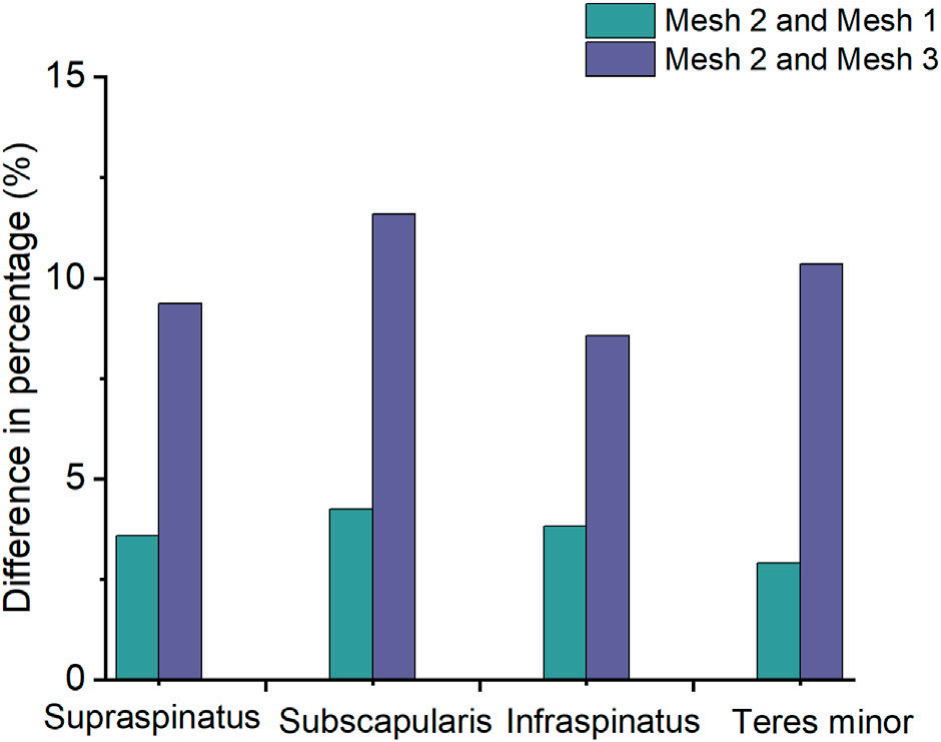
Difference in the percentage of the maximum von Mises stress between mesh 2 and mesh 1 and between mesh 2 and mesh 3 in the rotator cuff during shoulder abduction.

To test mesh convergence on the finite element shoulder model, mesh schemes were generated with element sizes of 0.5 mm (mesh 1), 1.0 mm (mesh 2), and 1.5 mm (mesh 3). A torque of 17.5 Nm was applied to the distal humeral surface for abduction, with six degrees of freedom constrained at the proximal clavicle and the inferior surface of the scapula. The maximum von Mises stresses in the rotator cuff tissue were calculated and compared in the finite element model. The mesh was considered convergent if the difference in the results between the smallest unit size and other mesh sizes compared was less than 5% (Jones and Wilcox, 2008). The results, as shown in Figure 2, indicate that the stress value difference was less than 5% in mesh 1 compared to mesh 2. Therefore, the element size in the current model was 1 mm, with a total of 143,580 nodes.

### 2.3 Boundary conditions and simulation

The boundary conditions and mechanical load conditions of this study were based on previous studies (Zheng et al., 2020; Asadi Dereshgi and Serbest, 2022). Loads of 0 kg (non-load condition) and 7.5 kg (load condition) were separately applied to the intercondylar center of the humerus based on the shoulder finite element model to simulate the effects of different loads on the shoulder tissues. The lower part of the scapula and the proximal clavicle were kept fixed. The contact between the rotator cuff tissue and the humerus was set as bound, and the articular cartilage was set as frictionless (Zheng et al., 2020). For glenohumeral abduction and adduction, an additional moment of 17.5 Nm was applied to the humerus (Islán Marcos et al., 2019). The aforementioned load conditions were imported into LS-DYNA software for calculation. The maximum von Mises stress distributions of the rotator cuff and average stress in the supraspinatus tendon at 0, 15, and 30° of glenohumeral abduction and adduction were recorded and analyzed to investigate the effects of load on the rotator cuff tissue with different motions.

## 3 Results

### 3.1 Stress results of the supraspinatus

**FIGURE 3:**
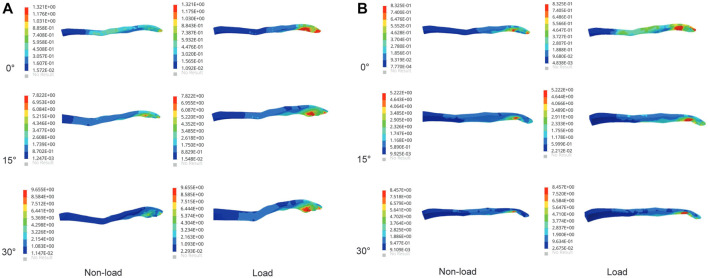
Maximum von Mises stress of supraspinatus muscle under non-load and load conditions. **(A)** Maximum von Mises stress of the supraspinatus during shoulder abduction. **(B)** Maximum von Mises stress of the supraspinatus during shoulder adduction.

**FIGURE 4:**
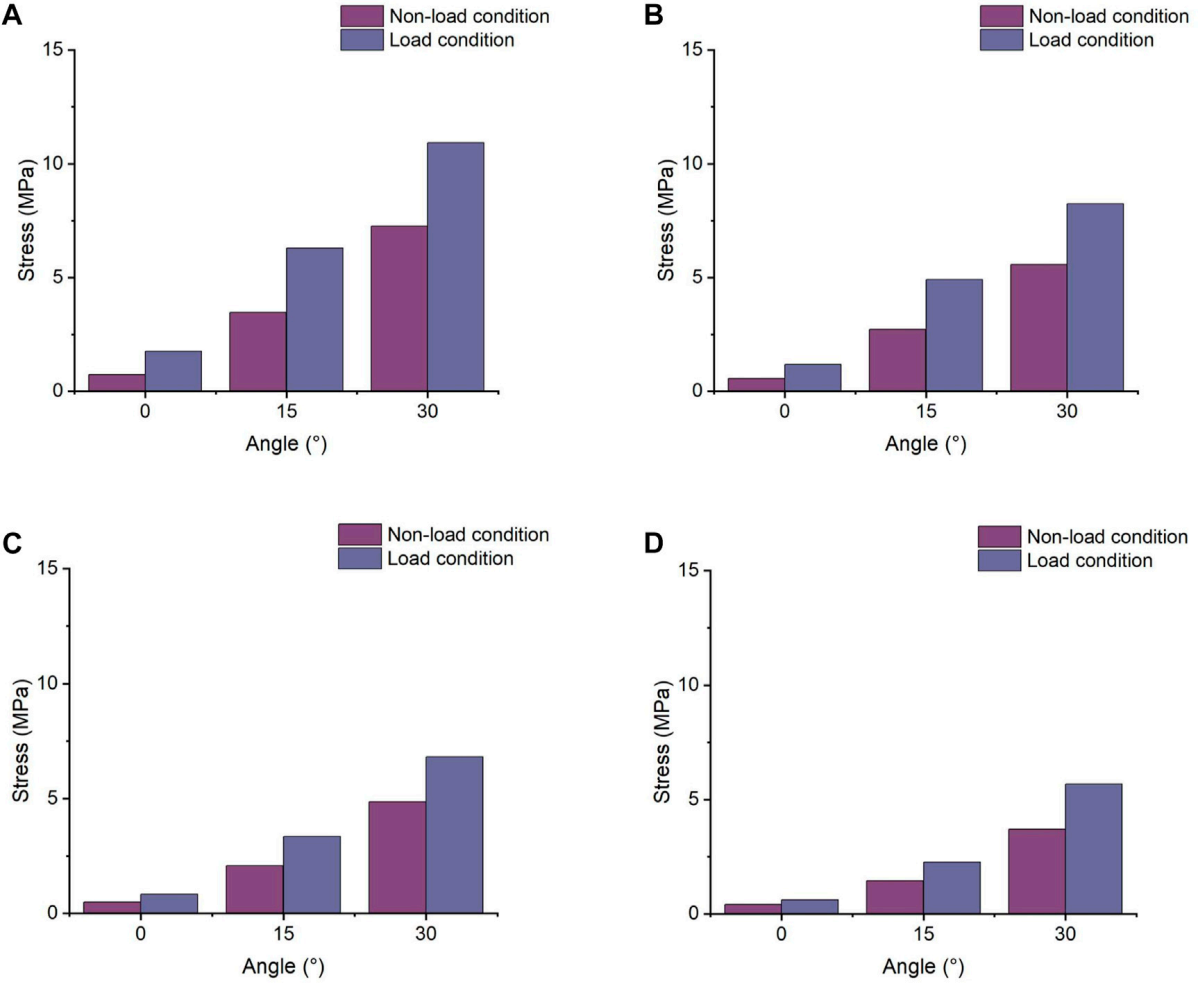
Comparison of the average joint side and bursal side stress in the supraspinatus. **(A)** Stress of the articular side during shoulder abduction. **(B)** Stress of the bursal side during shoulder abduction. **(C)** Stress of the articular side during shoulder adduction. **(D)** Stress of the bursal side during shoulder adduction.

The results, as shown in Figure 3, indicate that the maximum von Mises stresses in the supraspinatus tendon increased under both abduction and adduction conditions. The stresses under the load and non-load conditions were 1.32 and 3.35 MPa at 0° abduction, respectively. At 30° abduction, the load condition experienced 75% more stress in the supraspinatus than the non-load condition. Compared to the non-load condition, the load condition had a 72% increase in supraspinatus stress at 0° adduction, while at 30°, the load condition had a 26% increase. Figure 4 shows that the supraspinatus tendon has a greater average stress on its joint side than its bursal side, causing the difference in stress. The joint and bursal average stresses at 30° abduction were 10.92 and 8.24 MPa under the load condition, respectively, which showed a difference of 32% between the two sides.

### 3.2 Stress results of the subscapularis

**FIGURE 5:**
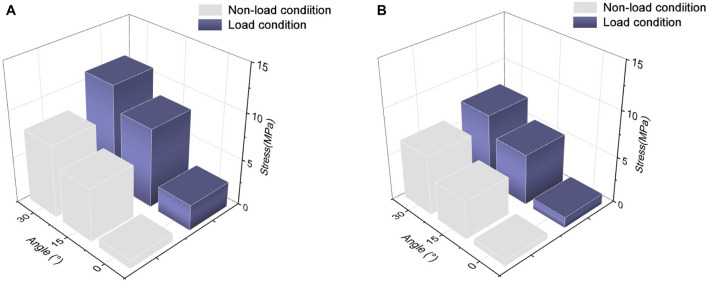
Maximum von Mises stress changes in the subscapularis muscle. **(A)** Stress variation in the subscapularis muscle under shoulder abduction. **(B)** Stress variation in the subscapularis muscle under shoulder adduction.

The stress value of the subscapularis increased under both conditions at 15° and 30° abduction, as shown in Figure 5, with the maximum stress not exceeding 12 MPa. The stress values at 15° and 30° adduction under the load condition were 5.53 and 7.65 MPa, respectively. Compared with the non-load condition, the subscapular muscle stress increased by 42% and 19% under the load condition during abduction 30° and adduction 30°, respectively.

### 3.3 Stress results of the infraspinatus

**FIGURE 6:**
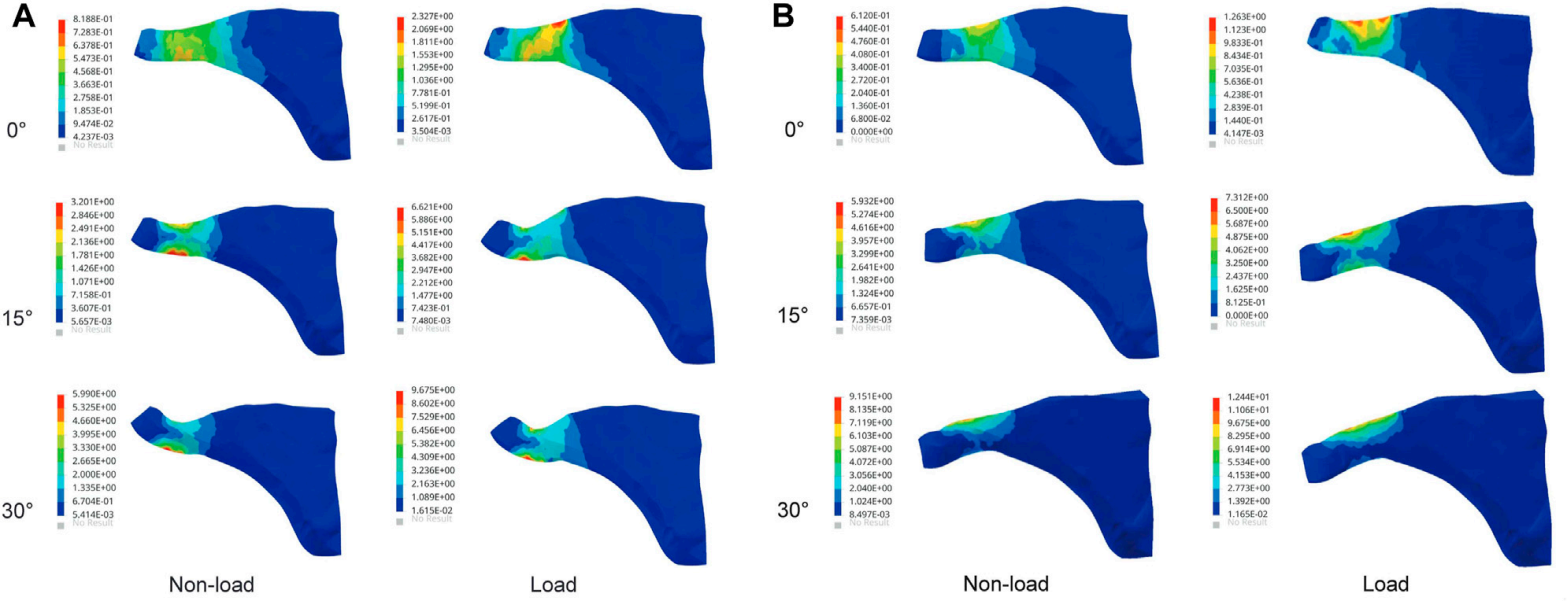
Maximum von Mises stress results of infraspinatus muscle. **(A)** Stress variation in the infraspinatus muscle during shoulder abduction. **(B)** Stress variation in the infraspinatus muscle during shoulder adduction.

The maximum von Mises stress values of the infraspinatus were 5.99 and 9.67 MPa under the non-load and load conditions at 30° abduction, respectively, indicating a 61% increase in stress under the load condition compared to the non-load condition. At 30° adduction, the stresses in the infraspinatus muscle were 9.15 and 12.44 MPa under the non-load and load conditions, respectively, with a difference of 36%. As shown in Figure 6, the infraspinatus tendon–humerus junction was the area of the humerus where the stress was concentrated.

### 3.4 Stress results of the teres minor

**FIGURE 7:**
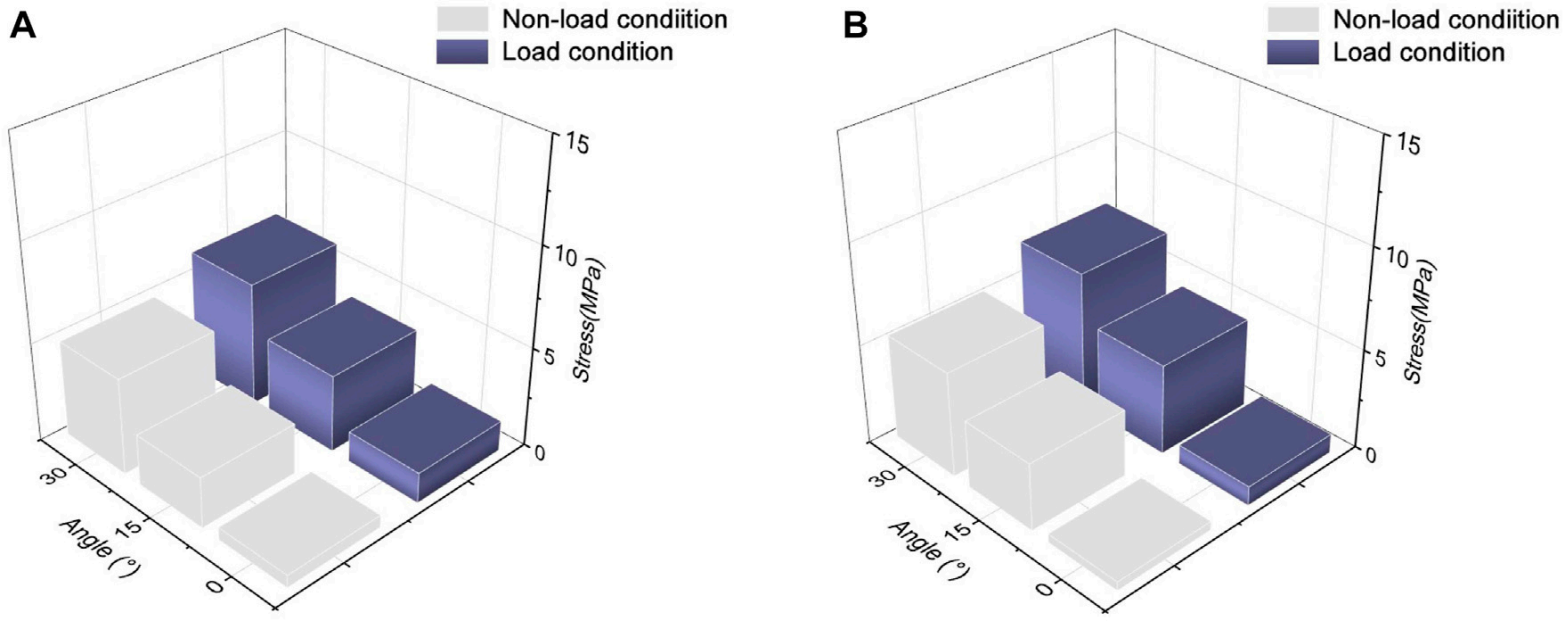
Maximum von Mises stress of the teres minor muscle. **(A)** Maximum von Mises stress of the teres minor during shoulder abduction. **(B)** Maximum von Mises stress of the teres minor during shoulder adduction.

At 0° abduction, the two conditions had a stress of 0.63 and 1.54 MPa, respectively, on the teres minor. The stress under the load condition was 51% and 33% higher than that under the non-load condition at 15° abduction and adduction, respectively. Figure 7 shows that the load condition experienced higher stress than the non-load condition during glenohumeral movement. The maximum stress values under the load condition reached 6.24 and 6.87 MPa at 30° abduction and adduction, respectively.

## 4 Discussion

Rotator cuff muscles are divided into subscapularis, supraspinatus, infraspinatus, and teres minor muscles, which provide stability to the shoulder joint (Medina et al., 2021). Each shoulder movement is completed by the combined action of muscles and joints. Movement of the shoulder causes a redistribution of stress in the shoulder tissues, and excessive or prolonged mechanical stress can increase degeneration and injury to the rotator cuff tissues (Rodriguez Diez-Caballero et al., 2020). Therefore, analyzing the mechanical distribution of shoulder tissues resulting from changes in the load can help explain the biomechanical factors that cause rotator cuff injuries.

Epidemiological studies of rotator cuff injuries showed that the supraspinatus muscles were a common type of rotator cuff injury (Xu et al., 2020), which may be due to tissue degeneration as a result of prolonged repetition of certain movements and sustained loads (Pozzi et al., 2022; Riddervold et al., 2022). Our results showed that higher stress was concentrated in the supraspinatus during abduction and in the infraspinatus during adduction. The supraspinatus muscle plays an abductor role in the shoulder joint. The load condition was found to have more rotator cuff tissue stress than the non-load condition. Activation of the myoelectric signals in the rotator cuff tissues increases the stability of the shoulder joint under load conditions (Reed et al., 2016). Andersen et al. (2010) found that load exercises require greater activation of rotator cuff tissue forces and are more likely to result in muscle fatigue. The increase in load changes the mechanical distribution of the rotator cuff tissues, which increases the local tissue stress concentration.

A stress nephogram revealed that the maximum stress in the rotator cuff was near the tendon–humeral junction, which was consistent with the common site of injury to the rotator cuff (Inoue et al., 2013). Compared to the bursal side, supraspinatus muscle stresses increased by up to 32% on the joint side. The difference in stress between the joint and bursal sides was one of the main causes of supraspinatus injury. A tensile test of the supraspinatus tendon conducted by Huang et al. (2005) revealed mechanical differences between the joint and bursal sides, with the joint side having a higher mechanical index than the bursal side. One possible injury factor was the irregular arrangement of collagen in the articular side of the supraspinatus muscle compared with the more regular arrangement of collagen in the bursal layer of the rotator cuff tendon (Nakajima et al., 1994). A comparison of the load and non-load conditions showed that the load condition increased the stress concentration in the supraspinatus during shoulder movement and increased the stress difference between the joint side and the bursal side. Rotator cuff injuries were associated with repetitive loads (Pozzi et al., 2022), but there is still no specific numerical definition of repetitive load, which requires further experimental study.

In this study, rotator cuff tissue stresses were used to reflect the biomechanical changes in shoulder tissue under load. Under the abduction and adduction conditions, the rotator cuff tissue stresses showed different degrees of increase and stress concentration under the non-load and load conditions. However, the load condition further increased the stresses in the rotator cuff tissues and the stress difference between the supraspinatus joint side and bursal side stress compared to the non-load condition and changed the mechanical distribution of the rotator cuff tissues.

The current study has some limitations. The shoulder model was constructed based on imaging data from only one normal individual, and more studies should be included in future cohort studies. The shoulder model lacks other structures such as the bursa, biceps, and triceps. A more detailed model needs to be constructed in further studies. The muscle belly becomes bigger when it activates *in vivo*, which could not be modeled in the finite element simulation directly. The active muscular action of the muscle is not considered, but it is treated as a passive structure. It requires multidisciplinary cooperation in the future to build material parameters that are more consistent with the human body. In the validation section, we only analyzed the tension of the supraspinatus muscle and the validated metrics correlated with the result, while we did not validate the mechanical changes in other rotator cuff tissues due to the lack of previous literature. This study examined only the mechanical changes in the rotator cuff tissues during abduction and adduction, and subsequent simulation analysis should consider more movements and other mechanical outcomes of shoulder tissues.

## 5 Conclusion

The present study assessed the mechanical effects of shoulder load on the rotator cuff tissue using a finite element model. We found that increased loads changed the mechanical distribution of the rotator cuff tissues and increased the stress difference between the supraspinatus joint and the bursal side. In summary, increased load leads to stress concentrations in certain rotator cuff tissues, which may lead to tissue injury.

## Data Availability

The original contributions presented in the study are included in the article/Supplementary Material; further inquiries can be directed to the corresponding author.
